# Patterns of engagement in digital mental health intervention for LGBTQ+ youth: a latent profile analysis

**DOI:** 10.3389/fdgth.2023.1254929

**Published:** 2023-11-23

**Authors:** Seul Ki Choi, Emma Bruehlman-Senecal, Amy Green, Josh Lavra, José Bauermeister

**Affiliations:** ^1^ Department of Family and Community Health, School of Nursing, University of Pennsylvania, Philadelphia, PA, United States; ^2^ Hopelab, San Francisco, CA, United States

**Keywords:** digital health intervention, engagement, *paradata*, stigma, minority stress, mental health, LGBTQ+ youth

## Abstract

Engagement is a key metric that researchers can use to assess whether participants received the intended dose of a digital health intervention. However, the prevailing approach has predominantly focused on individual *paradata* metrics, resulting in a fragmented understanding of overall engagement. To address this limitation, our study utilizes person-centered approaches that allow for the simultaneous capture of multiple engagement metrics within *imi–*a web application specifically designed to support the mental health of lesbian, gay, bisexual, transgender, queer, and other sexual and gender minority youth (LGBTQ+ youth). This person-centered approach enabled us to explore the association between engagement patterns and stress appraisal outcomes within the *imi* intervention arm. Utilizing latent profile analysis, we classified users into two engagement forms: *overall engagement* (total number of sessions, pages visited, and external links clicked and their cumulative time spent using *imi*) and *content engagement* (number of pages viewed across *imi*'s four core guides: gender, stress, queerness, and stigma). We identified two profiles for each form: a “high engagement” profile and an “average engagement” profile, with the majority of participants assigned to the “average engagement” profile. Our analyses revealed a significant association between *overall engagement* profiles and stress appraisals, with the “high engagement” profile demonstrating higher challenge appraisals and marginal improvements in threat appraisals compared to the “average engagement” profile. However, no such associations were observed for *content engagement* profiles and stress appraisal outcomes. The two person-centered approaches used were consistent with prior results utilizing a variable-centered approach, indicating a stronger intervention effect among individuals who exhibit higher engagement in digital health interventions. Although both methods yielded comparable findings, the person-centered approach mitigates concerns related to multi-collinearity and adds additional nuance and context to the study of digital engagement.

## 1. Introduction

Digital health interventions (DHIs) have become increasingly popular in recent years as a means of promoting healthy behaviors and reducing healthcare costs through their affordability and accessibility. Particularly for younger generations, DHIs have proven to be an excellent resource for improving health outcomes (1). DHIs also can be tailored to meet the particular needs of individuals, making them a promising tool for enhancing health outcomes (2, 3). Furthermore, DHIs have proven to be highly effective, especially within sexual and gender minority (SGM) communities, as DHIs serve as a valuable tool for reaching SGM youth (4–6). Despite their promising outcomes, the true effectiveness of DHIs is a matter of concern. A review of 24 unique DHIs seeking to address mental, physical, or sexual health–related concerns in lesbian, gay, bisexual, transgender, queer, and other sexual and gender minority (LGBTQ+) young people found the methodologies to be lacking in rigor and the quality of evidence to be poor (7). Researchers have noted that the variable efficacy across trials may be dependent on participant engagement with the interventions (8), as opposed to their reach or acceptability.

Engagement can be understood as a multi-faceted concept encompassing three fundamental components—behavior, cognition, and affect—that are commonly shared across various domains (9, 10). Considering the wide variability in engagement with previous DHIs (11), analyzing engagement to assess whether participants received the intended dose of a DHI becomes a crucial effectiveness and implementation outcome. Previous studies that have examined the relationship between engagement and outcomes suggest that engagement positively predicts improved health outcomes (11). A recent meta-analysis of DHIs focused on mental health found greater engagement to be positively associated with therapeutic gains (12). Similarly, greater engagement has been associated with changes in other health domains (e.g., HIV prevention and care). For example, in an online intervention to reduce sexual risk behaviors among young Black men who have sex with men, researchers found a stronger intervention effect among those who used the intervention for more than 60 min over the 3-month intervention period (8).

The behavioral aspect of engagement, often referred to as “usage,” is the most frequently employed dimension. *Paradata* (i.e., automatically generated process data that captures participants' actions within an application or website) allows for nuanced and interrelated explorations regarding how users' behavioral engagement within a DHI are associated with an intervention's outcomes (13–16). Using *paradata*, Hightow-Weidman and Bauermeister characterized engagement metrics into four domains: amount, duration, frequency, and depth. This characterization offers a foundation for standardizing the use of *paradata* to examine behavioral engagement in DHIs (11). To date, however, there has been a tendency to explore individual *paradata* metrics (i.e., amount, duration, and depth of use) as independent predictors of outcomes in DHI analyses (11). For example, Choi et al. explored the correlation of engagement within an online HIV prevention intervention for single young men who have sex with men using two individual *paradata* metrics; the number of log-ins and the number of sessions viewed (17). Also, Bonett utilized two individual *paradata* metrics, the time spent on the intervention components and interactions with features, to examine whether *paradata* metrics differed by participants' characteristics in a brief online intervention to reduce barriers to healthcare for young men who have sex with men (18). However, these traditional variable-centered approaches, which focus on the predictive power of individual *paradata* metrics, have limitations in explaining the complex nature of user engagement within DHIs (4, 11) and often result in multiple testing issues, including inflated Type I error rates (19, 20). Therefore, there is a need to examine the utility of alternative methodologies like person-centered approaches (i.e., mixture cluster analysis, latent profile analysis, or latent class analysis) to recognize the multidimensional nature of engagement (21) and correct for these statistical challenges.

Person-centered approaches are extensively utilized in human behavior research as they enable researchers to gain a comprehensive understanding of complex behaviors by considering multiple factors simultaneously (22, 23). Moreover, the person-centered approach can help address multi-collinearity issues that may arise from the variable-centered approach. In the context of DHI engagement, there are various metrics (i.e., amount, depth, and frequency) that contribute to the quality of engagement beyond just duration (e.g., time spent), which is commonly used. Through the adoption of a person-centered approach, researchers can identify unmeasured patterns based on the distribution of *paradata* metrics concurrently. While a few studies have employed traditional variable-centered approaches to explore individual *paradata* metrics in DHIs (8, 13, 17, 24), there have been no previous studies that have utilized person-centered approaches to comprehensively understand engagement patterns in DHIs with multiple factors simultaneously. Therefore, it is crucial to investigate the utility of employing a person-centered approach in examining DHI engagement, as this may offer new insights and facilitate a more accurate understanding of engagement.

We utilized *imi,* a web application (web-app) designed to facilitate LGBTQ+ identity affirmation for teens and young adults (ages 13–19), as a case study (25). The *imi* application was created with the aim of fostering affirmation of SGM identities, fostering a sense of belonging within the LGBTQ+ community, and motivating the practice of cognitive and behavioral coping skills. Consistent with the Minority Stress Model (26), *imi* recognizes that addressing the well-being of LGBTQ+ youth must acknowledge that negative health outcomes often arise from a hostile culture towards sexual and gender minorities, which creates stressors unique to minority identity, including harassment, victimization, internalized homophobia, and expectations of rejection. To address these challenges, researchers (27–30) have argued that LGBTQ+ interventions should focus on providing resources for coping with minority stress, including strengthening SGM youth's ability to cope, teaching additional cognitive and behavioral coping skills, affirming SGM identities, and strengthening supportive social connections. Therefore, the *imi* web-application offered content, interactive activities, stories, images and videos from LGBTQ+ youth across four content areas: (1) exploring gender identity (gender), (2) exploring sexual orientation and broader LGBTQ+ identities (queerness), (3) managing externalized SGM stressors (e.g., discrimination) and learning coping skills (stress), and (4) dealing with internalized homophobia and transphobia (stigma). Results from the *imi* trial (25) supported the value of this approach, with participants assigned to the intervention arm reporting improved challenge appraisals (i.e., belief in one's coping abilities) than participants in the attention-control arm. Moreover, main outcome results found that specific individual *paradata* metrics (≥5 sessions, >10 min, or >10 pages) were associated with greater benefits among *imi* intervention arm participants (25).

In this analysis, we used a person-centered approach to characterize the *paradata* collected in the *imi* intervention and examine whether different typologies of engagement are associated with participants' stress appraisals. We proposed two person-centered approaches: an *overall engagement* approach and a *content engagement approach*. The *overall engagement* approach explores how users interact with a DHI by employing four behavioral engagement indicators: (1) users' cumulative time spent on *imi*, (2) their total number of sessions, (3) pages visited, and (4) external links clicked. The *content engagement* approach, on the other hand, examines participants' number of page views across the four topic-specific guides within *imi*: (1) gender, (2) stress, (3) queerness, and (4) stigma. The *content engagement* approach allowed for an assessment of whether individual differences in the specific nature of the content consumed were predictive of the benefits participants derived from the intervention. Using these two distinct forms of engagement (*overall engagement* and *content engagement*), we examined the relationship between engagement profiles and changes in stress appraisals, which is the primary outcome of the *imi* study, across the course of the 4-week intervention (25). We also compare our person-centered approach results with those previously reported using the traditional variable-centered approach.

## 2. Materials and methods

Data for this analysis came from a pilot randomized controlled trial (RCT) of *imi*, a web application designed to improve mental health by supporting lesbian, gay, bisexual, transgender, queer, and other LGBTQ+ youth identity affirmation, coping self-efficacy, and coping skills. A detailed procedure for the *imi* study has been previously outlined elsewhere (25).

### 2.1. Study procedures

**Figure 1:**
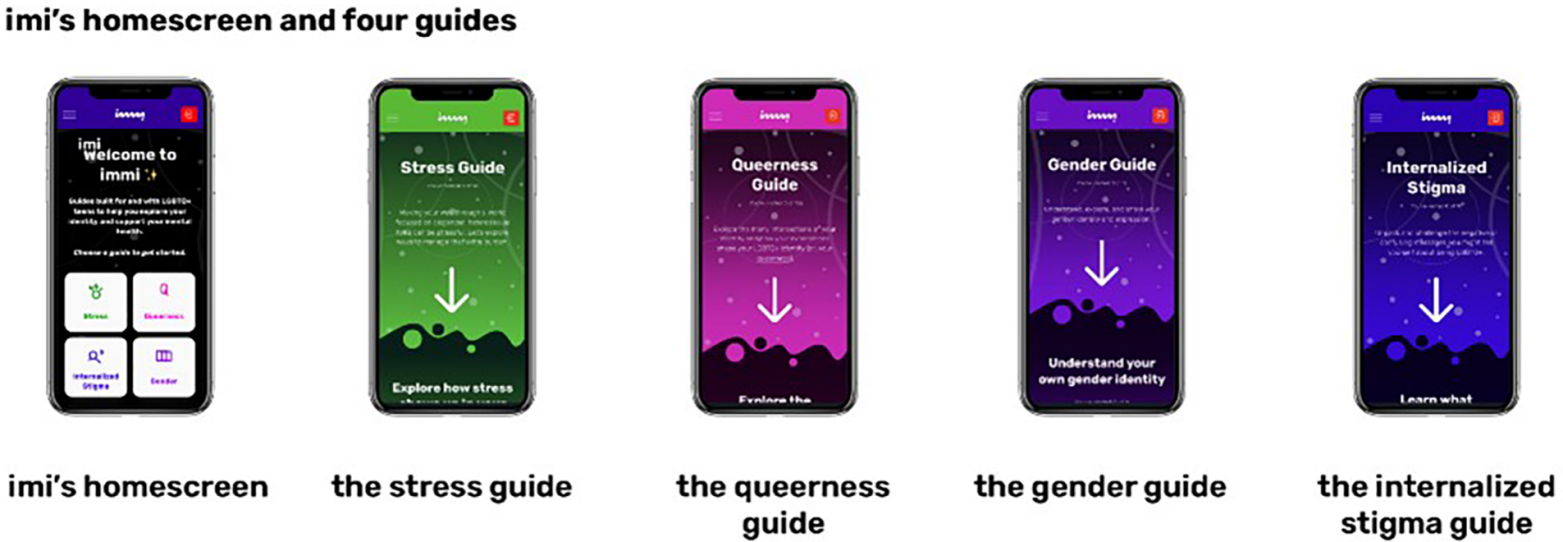
Screenshots from the *imi* (treatment) web applications [from Bauermeister et al. (25)].

This is a 4-week pilot RCT to evaluate the efficacy of the *imi* web-application. Participants were randomized to either the intervention arm (called “*imi*”), which has full access to the application or the control arm which had access to curated resource pages which were mirrored on *imi* (see Figure 1; currently accessible at https://imi.guide/). We collected survey data via web-based self-completed Qualtrics surveys administered at baseline and a 4-week follow-up. Participants' actions in the application were collected as *paradata*. Participants were recruited across the United States through advertisements on Instagram. Participants had to (1) be 13 to 19 years of age (inclusive); (2) self-identify as LGBTQ+; (3) reside within the United States; (4) have English literacy; (5) have access to a device with internet access, a web browser, and SMS text messaging capabilities; and (6) be willing to participate in study activities for 4 weeks. To ensure that our results generalize to racial/ethnic minority LGBTQ+ youth, who often face multiple forms of intersecting minority stress, we over-recruited racial/ethnic minority participants such that they comprise approximately 75% of our sample.

Interested participants completed an online screener between October and November 2021. If eligible, they were shared an email link to the consent form and baseline survey. Participants had two weeks to complete the survey. 162 of the 488 screeners who were invited to consent and complete the baseline survey did not participate during the allowable two-week period. In addition to established best practices for participant verification (31–33), we compared participants’ screener data to their baseline responses across various questions asked on both assessments. If inconsistencies were identified, individuals were emailed and asked to respond via email or phone to resolve the issue. 56 participants did not pass the participant verification procedure (i.e., there were discrepancies between information entered on screener and baseline surveys). In total, a sample of 270 LGBTQ+ youth (mean age 16.49; SD 1.49) was enrolled and randomized into the intervention arm and control arm in a 1:1 fashion.

### 2.2. Intervention description

The *imi* web-application is a tool that provides curated content, interactive activities, videos, stories, and imagery from LGBTQ+ youth across guides covering four content areas. Content was tailored based on youth feedback and contributions (i.e., interviews, focus group, and co-design sessions). These areas are: (1) exploring gender identity (gender), (2) exploring sexual orientation and broader LGBTQ+ identities (queerness), (3) managing externalized SGM stressors (e.g., discrimination) and learning coping skills (stress), and (4) dealing with internalized homophobia and transphobia (stigma). Participants in the intervention group also had access to resource webpages that linked to the additional crisis and non-crisis resources, which were also available to the control group. Participants were asked to use *imi* at least once per week.

### 2.3. Ethics approval

All study procedures were approved by the Institutional Review Board at the University of Pennsylvania (protocol 849509), and the study was registered on ClinicalTrials.gov (NCT05061966). We received a waiver of parental consent for youth who may not have disclosed their gender identity or sexuality to their parents or who may have limited parental support to participate in the study.

### 2.4. Measures

#### 2.4.1. Engagement profiles

This study analyzed the intervention arm (*n* = 135) *paradata* over the 4 week intervention period and the main outcome at the end of 4 weeks. Over the 4 week intervention period, participants' actions in *imi* were collected as *paradata*. *Paradata* can be transformed to characterize the amount, frequency, duration, and depth of engagement with a web-based intervention. In this study, we present two distinct forms of engagement, (1) *overall engagement* and (2) *content engagement* to characterize their potential to inform the evaluation of DHIs in the future.

##### 2.4.1.1. Overall engagement

We derived four *paradata* metrics to define *overall engagement*: (1) counts of user sessions, (2) time spent in the intervention, (3) the number of pages visited, and (4) external links clicked.

##### 2.4.1.2. Content engagement

We also derived the number of pages visited across theoretically-anchored guides: (1) gender identity exploration (gender), (2) sexual orientation and broader LGBTQ+ identity exploration (queerness), (3) stress and coping (stress), and (4) internalized homophobia and transphobia (stigma).

#### 2.4.2. Primary outcomes

The primary outcome in the *imi* RCT was stress appraisals. The Stress Appraisal Measure for Adolescents (34) captures stress appraisals across 3 dimensions (challenge, threat, and resources). The 3-item Challenge subscale assesses perceptions of stress as a surmountable challenge (Cronbach α = .67). The 7-item Threat subscale measures perceptions of stress as having lasting, negative repercussions (α = .83). The 3-item Resources subscale assesses the belief that one has the necessary internal and external resources to cope with stress (α = .81). Responses to all items are recorded on a 5-point scale (1 = strongly disagree to 5 = strongly agree). A mean score was computed for each subscale, with higher values indicating greater endorsement of each respective stress appraisal.

#### 2.4.3. Demographic characteristics

We asked the participants to report characteristics regarding their age, race, ethnicity, highest level of education, subjective socio-economic status, gender identity, and sexual orientation. For gender identity and sexual orientation, respondents were given the option to select multiple identities. Subsequently, we categorized gender identity as either “mutually exclusive cisgender” or “other.” Similarly, we dichotomized sexual orientation into “single identity” or “multiple identities.” Further details about non-mutually exclusive patient characteristics can be found elsewhere (25).

### 2.5. Statistical analysis

Descriptive statistics were used to summarize the study participants' demographic characteristics and engagement characteristics, including total session completed, total time spent, time of engagement, engagement devices (phone or computer), unique page viewed, and the number of links clicked.

We utilized latent profile analysis to determine the number of engagement profiles (35). For *overall engagement*, we used the z-scores for four sub-constructs: (1) counts of user sessions, (2) time spent on each intervention, (3) the number of pages visited, and (4) external links clicked. For *content engagement* profiles, we used the mean score for four sub-constructs: (1) the number of pages visited in the gender guide, (2) the number of pages visited in the queerness guide, (3) the number of pages visited in the stress guide, and (4) the number of pages visited in the stigma guide. The most appropriate model was chosen using several criteria, including the Akaike Information Criteria (AIC), Bayesian Information Criteria (BIC), Vuong-Lo-Mendell-Rubin Likelihood Ratio Test (LRT), and entropy (36). Lower AIC and BIC scores indicate a better model fit, and a higher entropy score indicates better class separation. A significant LRT between the k-1 profile model and the k profile model indicates that the model fit improved from the former to the latter. Once the best-fitting model was selected, each profile was defined based on the mean scores of the four sub-constructs. Latent profile analysis was conducted using Mplus version 8 (37). After finalizing the best-fitting model, Cohen's kappa coefficient was used to evaluate the degree of agreement between the two engagement profiles (38).

Finally, we used linear regression to test the main effect of engagement (latent engagement profiles) on primary outcomes at week 4, adjusting for the baseline value of each respective outcome as a covariate. We also tested for changes over time within each engagement profile. We used SAS 9.4 to examine the associations between engagement profiles and primary outcomes (Cary, NC: SAS Institute Inc.).

## 3. Results

### 3.1. Sample characteristics

Participants (*n* = 135) had a mean age of 16.6 (SD 1.46) years. Most participants lived in a metropolitan area (91.1%) and identified as racial/ethnic minorities (76.3%). Over a third of participants identified with multiple genders (42.2%) and sexual identities (39.3%).

### 3.2. Engagement

Users completed a median of four sessions, viewed 25 unique pages (the maximum number of unique pages for *imi* was 73), and spent 11.6 min in *imi*. More than half of the participants accessed *imi* between 9am and 6pm. Participants had the option to access the web-app using either mobile phones or computers, and it was observed that a higher percentage of participants (81.1%) chose to use mobile phones, while a smaller percentage (38.6%) used computers (not mutually exclusive; some participants had access to both). Given external links were the sole component of the control arm whereas they were embedded within a lot of other content within the intervention arm, the median number of links clicked was 0 (range 0–6).

### 3.3. Latent profile analysis

#### 3.3.1. Overall engagement

**Figure 2:**
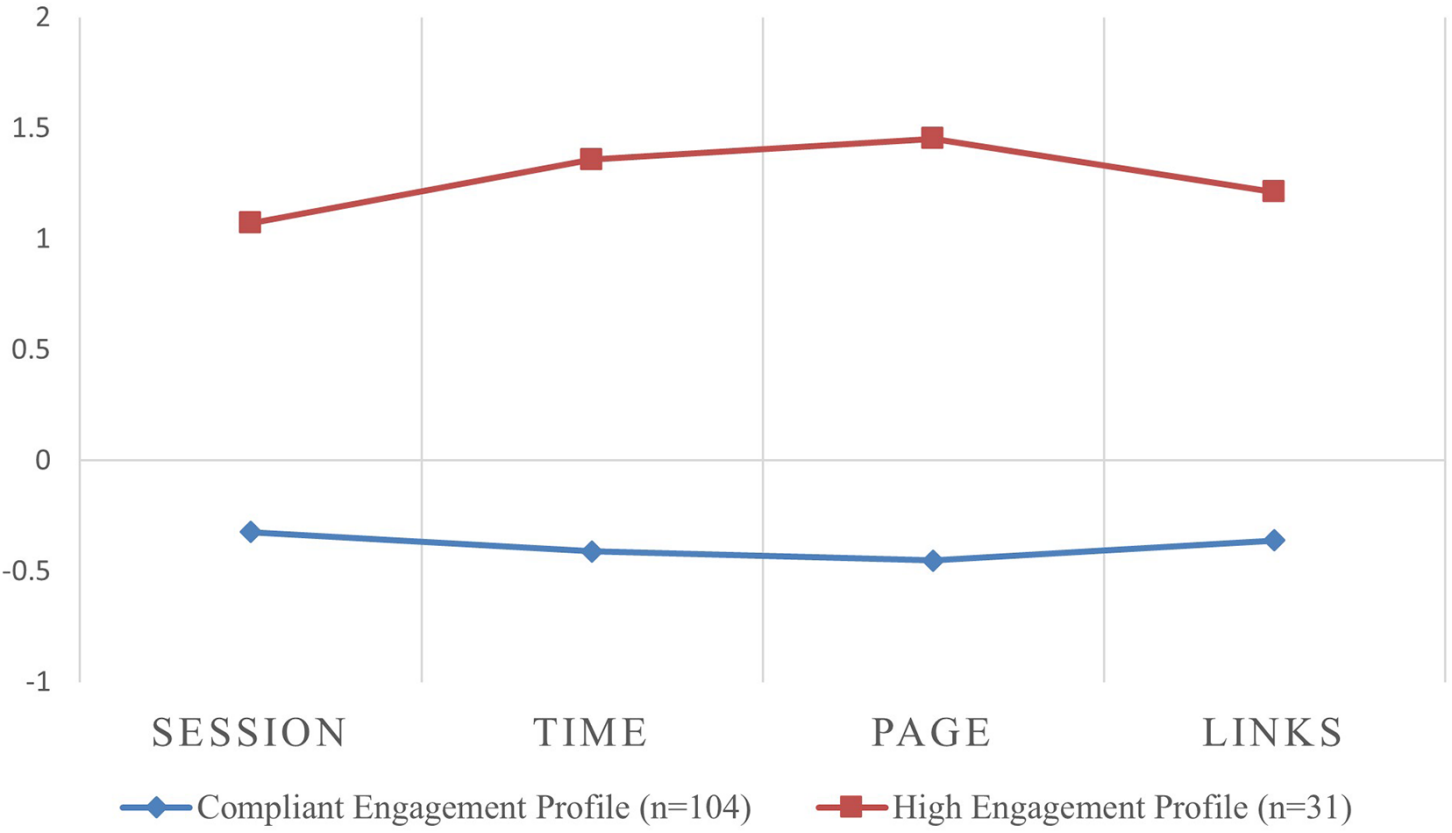
Mean *Z*-scores for the four engagement metrics by overall engagement profiles. Session: total number of sessions; Time: cumulative time spent; page: total number of pages visited; links: total number of external links clicked.

We iteratively compared models with increasing numbers of profile solutions using AIC and BIC. AIC and BIC get smaller as the number of profiles increases. Entropy was similar across the different solutions. The difference in LRT between a 2-class solution and a 3-class solution was not statistically significant (two-times-the-log likelihood difference = 133.48, df = 5, *p* = 0.166) (see Table 1). Therefore, a 2-class latent profile solution was selected from the empirical and theoretical perspective as the optimal model; the results of the 2-profile solution are shown in Figure 2. The “average engagement” profile represents the degree to which individuals reported mean engagement with *imi*. This profile accounted for the majority of the sample (*n* = 104; 77.0%) and was characterized by having comparable scores across the four derived *paradata* metrics (sessions, time, pages, and external links). The “high engagement” profile (*n* = 31; 22% of the sample) was characterized by being at least one standard deviation (range 1.06–1.97) above the mean for all metrics. Pages had the greatest difference (SD 1.91) between profiles, followed by external links (SD 1.81), minutes (SD 1.79), and sessions (SD 1.38).

**Table 1:** Fit indices for overall engagement profile analysis.

The number of profiles	AIC	BIC	Entropy	Vuong-Lo-Mendell-Rubin LRT	LRT—*p*-value
2 profiles	1,281.54	1,319.31	0.942	−755.763	<.0001
3 profiles	1,158.06	1,210.36	0.961	−627.770	0.166
4 profiles	1,093.78	1,160.61	0.966	−561.030	0.160
5 profiles	1,052.48	1,133.83	0.975	−523.892	0.176

AIC, akaike information criteria; BIC, Bayesian information criteria; LRT, likelihood ratio.

#### 3.3.2. Content engagement

**Figure 3:**
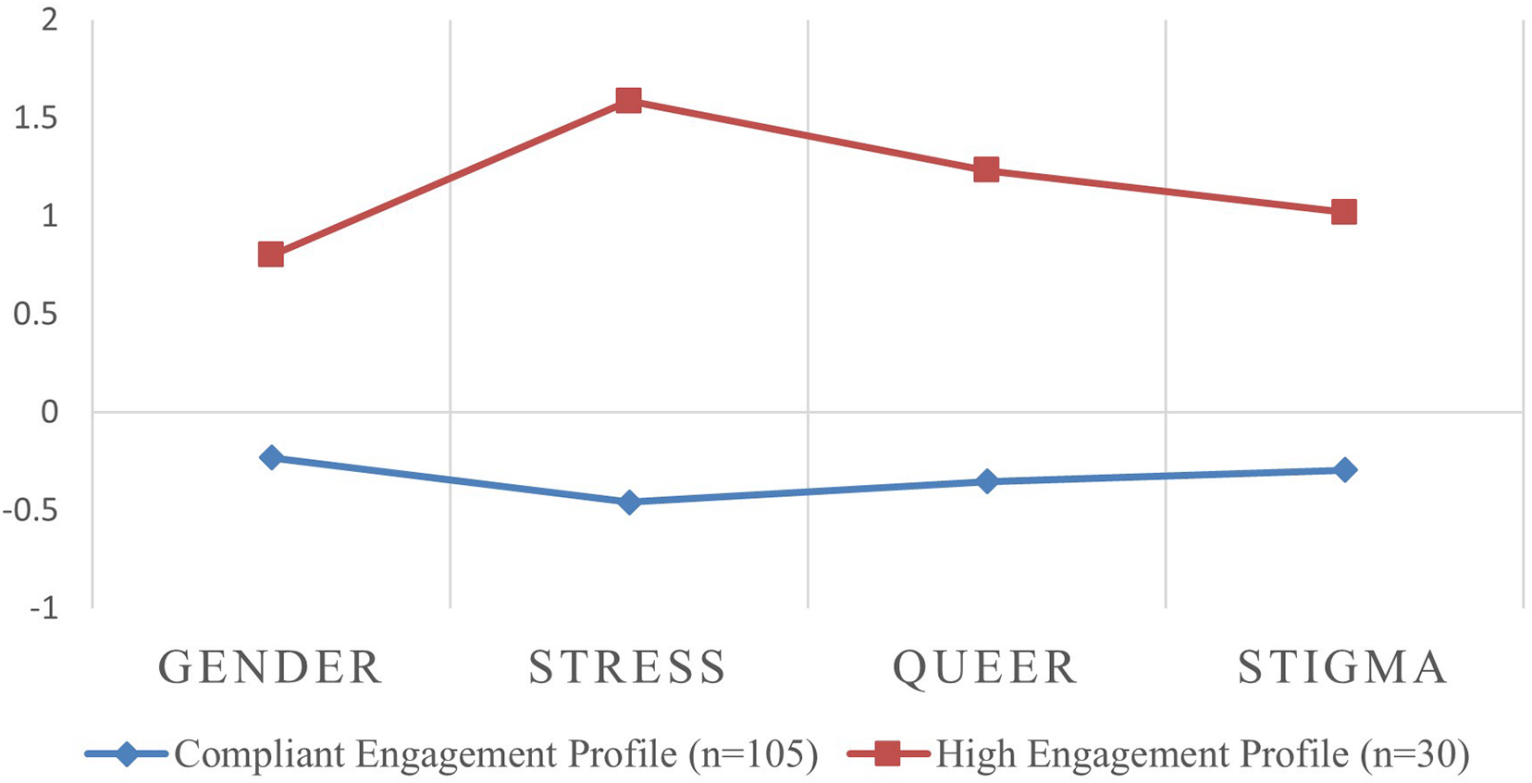
Mean *Z*-scores for the number of pages viewed in four guides by content engagement profiles. Gender: the number of pages visited in the gender guide; stress: the number of pages visited in the stress guide; queer: the number of pages visited in the queerness guide; stigma: the number of pages visited in the stigma guide.

Similar to the *overall engagement* results, analysis of *content engagement* showed that the values of AIC and BIC decreased with an increase in the number of profiles, indicating better model fit. Furthermore, the measure of model fit known as Entropy, remained consistent across the different solutions. The difference in LRT between a 2-class solution and a 3-class solution was not statistically significant (two-times-the-log likelihood difference = 199.1, *p* = 0.222; see Table 2), therefore, a 2-class latent profile solution was optimal for the *content engagement* (see Figure 3). The “compliant engagement” profile accounted for the majority of the sample (*n *= 105; 77.8%) and was characterized by comparable engagement across the four derived *paradata* metrics (gender, stress, queerness and stigma pages). Among four different content pages, participants read stress-related pages the least. The “high engagement” profile (*n *= 30; 21.2% of the sample) was characterized by around one standard deviation (range 0.83–1.59) above the mean for all metrics. The number of stress pages had the biggest difference (SD 2.05) between the two profiles.

**Table 2:** Fit indices for the content engagement profile analysis.

The number of profiles	AIC	BIC	Entropy	Vuong-Lo-Mendell-Rubin LRT	LRT—*p*-value
2 profiles	1,354.62	1,392.39	0.945	−763.860	<.0001
3 profiles	1,303.48	1,355.78	0.951	−664.310	0.222
4 profiles	1,262.57	1,329.39	0.899	−633.741	0.251
5 profiles	1,238.76	1,320.11	0.935	−608.286	0.300

AIC, akaike information criteria; BIC, Bayesian information criteria; LRT, likelihood ratio.

#### 3.3.3. Agreement between overall engagement profile and content profile

A Kappa agreement was employed to assess the level of agreement between the *overall engagement* profile and the *content engagement* profile. Classification of users between the *overall engagement* profile and the *content engagement* profile was high (Kappa = 0.81).

### 3.4. Engagement profiles, demographic characteristics, time of engagement, and modes of delivery

We examined the association between demographic characteristics and engagement profiles and none of the demographic characteristics differed by engagement profiles (see Table 3). We also analyzed time of engagement (i.e., the time of day in which participants' used *imi*) across the different engagement profiles (see Table 4). Participants in the “high engagement” profile, both for *overall engagement* and *content* profiles, exhibited greater engagement with the intervention during the time frames of 6am–9am and 6pm–12am local time, compared to the “compliant engagement” profile (*p* < 0.001). However, mode of delivery (phone vs. computer) was not associated with engagement profiles.

**Table 3:** Demographic characteristics by engagement profile (*N* = 135).

		Overall engagement			Content engagement		
	All (*N* = 135)	Average engagement profile (*n* = 104)	High engagement profile (*n* = 31)	*p*-value^a^	Average engagement profile (*n* = 105)	High engagement profile (*n* = 30)	*p*-value^a^
Age (years), mean (SD)	16.56 (1.5)	16.54 (1.5)	16.61 (1.4)	0.804	16.56 (1.5)	16.53 (1.4)	0.925
Geographic region, *n* (%)				0.563			0.567
Metropolitan	123 (91.1%)	94 (76.4%)	29 (23.6%)		95 (77.2%)	28 (22.8%)	
Micropolitan	7 (5.2%)	6 (85.7%)	1 (14.3%)		6 (85.7%)	1 (14.3%)	
Small town	3 (2.2%)	3 (100%)	0 (0%)		3 (100%)	0 (0%)	
Rural areas	2 (1.5%)	1 (50.0%)	1 (50.0%)		1 (50.0%)	1 (50.0%)	
Census region, *n* (%)				0.646			0.230
Northeast	21 (15.6%)	18 (85.7%)	3 (14.3%)		18 (85.7%)	3 (14.3%)	
Midwest	24 (17.8%)	17 (70.8%)	7 (29.2%)		15 (62.5%)	9 (37.5%)	
South	47 (34.8%)	37 (78.7%)	10 (21.3%)		38 (809%)	9 (19.1%)	
West	43 (31.9%)	32 (74.4%)	11 (25.6%)		34 (79.1%)	9 (220.9%)	
Education^a^, *n* (%)				0.822			0.844
K-5th grade	1 (0.7%)	1 (100%)	0 (0%)		1 (100%)	0 (0%)	
6–8th grade	50 (37.0%)	38 (76.0%)	12 (24.0%)		37 (74.0%)	13 (26.0%)	
9–11th grade	49 (36.3%)	36 (73.5%)	13 (26.5%)		38 (77.6%)	11 (22.5%)	
High school diploma	26 (19.3%)	22 (84.6%)	4 (15.4%)		22 (84.6%)	4 (15.4%)	
High school certificate of completion—no diploma	0 (0%)	-	-		-	-	
Some college, technical school, or vocational school	9 (6.7%)	7 (77.8%)	2 (22.2%)		7 (77.8%)	2 (22.2%)	
Two-year college graduate	0 (0%)	-	-		-	-	
Socio Economic Status, *n* (%)				0.238			0.297
Wealthy	0 (0%)	-	-		-	-	
Upper-middle class	25 (18.5%)	20 (80.0%)	5 (20.0%)		23 (92.0%)	2 (8.0%)	
Middle class	62 (45.9%)	49 (79.0%)	13 (21.0%)		47 (75.8%)	15 (24.2%)	
Working class	28 (20.7%)	20 (71.4%)	8 (28.6%)		19 (67.9%)	9 (32.1%)	
Low income or poor	12 (8.9%)	11 (91.7%)	1 (8.3%)		10 (83.3%)	2 (16.7%)	
I prefer not to respond	8 (5.9%)	4 (50.0%)	4 (50.0%)		6 (75.0%)	2 (25.0%)	
Ethnicity, *n* (%)				0.161			0.126
Hispanic	31 (23.0%)	21 (67.7%)	10 (32.3%)		21 (67.7%)	10 (32.3%)	
Non-Hispanic	104 (77.0%)	83 (79.8%)	21 (20.2%)		84 (80.8%)	20 (19.2%)	
Race, *n* (%)				0.202			0.358
Other	103 (76.3%)	82 (79.6%)	21 (20.4%)		82 (79.6%)	21 (20.4.%)	
White	32 (23.7%)	22 (68.8%)	10 (31.2%)		23 (71.9%)	9 (28.1%)	
Gender identity, *n* (%)				0.696			0.625
Other	112 (83.0%)	87 (77.7%)	25 (22.3%)		88 (78.6%)	24 (21.4%)	
Exclusive cisgender	23 (17.0%)	17 (73.9%)	6 (26.1%)		17 (73.9%)	6 (26.1%)	
Multiple sexual orientations, *n* (%)				0.943			0.451
Multiple identities	53 (39.3%)	41 (77.4%)	12 (22.6%)		43 (81.1%)	10 (18.9%)	
Single identity	82 (60.7%)	63 (79.8%)	19 (23.2%)		62 (75.6%)	20 (24.4%)	

^a^ Student *t* test for continuous variables and chi-square tests for categorical variables.

**Table 4:** Engagement metrics by engagement profile (*N* = 135).

Metrics	Overall (*n* = 135)	Overall engagement			Content engagement		
		Average engagement profile (*n* = 104)	High engagement profile (*n* = 31)	*P*-value^a^	Average engagement profile (*n* = 105)	High engagement profile (*n* = 30)	*P*-value^a^
Sessions							
Total sessions completed, median (25th pctl–75th pctl)	4 (2–7)	3 (2–5)	8 (6–10)	<.001	4 (2–5)	8 (3–10)	<.001
Time							
Total time spent (minutes), median (25th pctl–75th pctl)	11.60 (3.73–31.40)	6.76 (2.05–17.21)	57.98 (39.85–66.18)	<.001	6.77 (2.05–18.97)	58.31 (39.85–66.18)	<.001
Time of engagement^b^							
0:00am–before 3:00am	5 (3.8%)	3 (3.0%)	2 (6.5%)	0.335	2 (1.7%)	3 (10%)	0.077
3:00am–before 6:00am	21 (15.9%)	16 (15.8%)	5 (16.1)	1.00	14 (13.7%)	7 (23.3%)	0.256
6:00am–before 9:00am	45 (34.1%)	27 (26.7%)	18 (58.1%)	0.002	28 (27.5%)	17 (56.7%)	0.004
9:00am–before 12:00pm	75 (56.8%)	54 (53.5%)	21 (67.7%)	0.214	56 (54.9%)	19 (63.3%)	0.530
12:00pm–before 3:00pm	74 (56.1%)	52 (51.5%)	22 (71.0%)	0.065	52 (51.0%)	22 (73.3%)	0.037
3:00pm–before 6:00pm	72 (54.6%)	49 (48.5%)	23 (74.2%)	0.014	51 (50.0%)	21 (70.0%)	0.062
6:00pm–before 9:00pm	53 (40.2%)	32 (31.7%)	21 (67.7%)	<.001	33 (32.4%)	20 (66.7%)	0.001
9:00pm–before 0:00am	29 (22.0%)	17 (16.8%)	12 (38.7%)	0.014	18 (17.7%)	11 (36.7%)	0.043
Means of engagement^b^							
Phone	107 (81.1%)	84 (83.2%)	23 (74.2%)	0.298	84 (82.4%)	23 (76.7%)	0.596
Computer	51 (38.6%)	36 (35.6%)	15 (48.4%)	0.213	38 (37.3%)	13 (43.3%)	0.670
Unique pages^c^							
Unique pages viewed, median (25th pctl–75th pctl)	25.5 (11.5–63)	20 (8–34)	90 (76–113)	<.001	20 (8–37)	90.5 (76–113)	
Gender pages	2 (0–5)	1 (0–3)	7 (3–12)	<.001	1 (0–4)	5.5 (2–11)	<.001
Stress pages	2 (0–6)	2 (0–4)	11 (6–12)	<.001	2 (0–4)	12 (9–12)	<.001
Queer pages	2 (0–4)	1 (0–3)	7 (4–11)	<.001	1 (0–3)	9 (4–11)	<.001
Stigma pages	1 (0–3)	0 (0–2)	5 (2–9)	<.001	1 (0–2)	5 (2–9)	<.001
External links							
Number of links clicked, median (25th pctl–75th pctl)	0 (0–1)	0 (0–0)	2 (1–3)	<.001	0 (0–0)	1 (0–2)	<.001

^a^ Student *t* test for continuous variables and chi-square tests for categorical variables.

^b^ Compares dichotomized engagement time/means (engaged in a specific time frame vs. did not engage in a specific time frame) and engagement class (control vs. intervention).

^c^ The maximum number of unique pages for the *imi* application was 73.

### 3.5. Engagement profiles on primary outcomes

The observed associations between three stress appraisals and each engagement profile varied. For *overall engagement*, we identified a significant association with two stress appraisal outcomes: challenge appraisals and threat appraisals (see Table 5). The “high engagement” profile reported significantly higher challenge appraisals (Cohen *d* = 0.25; *p* = 0.034) compared to the “average engagement” profile. However, we did not find significant associations between *overall engagement* profiles and threat (Cohen *d* = −0.31; *p* = 0.057) and resource appraisals (Cohen *d* = 0.41; *p* = 0.086). None of the three stress appraisal outcomes were associated with the *content engagement* profiles (see Table 6).

**Table 5:** Changes in primary and secondary outcomes from baseline to the 4-week follow-up between overall engagement profiles.

Outcomes	Average engagement profile (*n* = 104)				High engagement profile (*n* = 31)					Modeling differences by engagement profile^b^	
	Baseline (*n* = 104), mean (SD)	Follow-up (*n* = 91), mean (SD)	*t* test (*df*)	*P* value^a^	Baseline (*n* = 31), mean (SD)	Follow-up, (*n* = 31), mean (SD)	*t* test (*df*)	*P* value^a^	Cohen *d*	Coefficients	*P*-value
Primary outcomes (Stress appraisals)											
Challenge	3.25 (0.77)	3.53 (0.79)	3.24 (90)	0.002	3.46 (0.74)	3.98 (0.95)	3.50 (30)	0.002	0.253	0.344	0.034
Threat	4.01 (0.69)	3.92 (0.68)	−1.70 (90)	0.093	3.91 (0.84)	3.61 (0.97)	−2.32 (30)	0.027	−0.308	−0.231	0.057
Resource	3.50 (0.98)	3.77 (0.94)	2.66 (90)	0.009	3.33 (0.98)	3.99 (0.87)	4.23 (3)	<.001	0.412	0.294	0.086

^a^ Paired *t* test.

^b^ The effect of engagement profile (average engagement vs. high engagement) on the outcome at follow-up controlling for outcome at baseline.

**Table 6:** Changes in primary and secondary outcomes from baseline to the 4-week follow-up between content engagement profiles.

Outcomes	Average engagement profile (*n* = 105)				High engagement profile (*n* = 30)					Modeling differences by engagement profile^b^	
	Baseline (*n* = 104), mean (SD)	Follow-up (*n* = 91), mean (SD)	*t* test (*df*)	*P* value^a^	Baseline (*n* = 31), mean (SD)	Follow-up, (*n* = 31), mean (SD)	*t* test (*df*)	*P* value^a^	Cohen *d*	Coefficients	*P* value
Primary outcomes (Stress appraisals)											
Challenge	3.23 (0.79)	3.54 (0.83)	3.40 (91)	0.001	3.54 (0.66)	3.98 (0.84)	3.59 (29)	0.001	0.128	0.292	0.078
Threat	4.02 (0.71)	3.91 (0.72)	−1.98 (91)	0.051	3.88 (0.79)	3.65 (0.90)	−2.03 (29)	0.052	−0.167	−0.148	0.232
Resource	3.48 (0.97)	3.75 (0.95)	2.80 (91)	0.006	3.42 (1.00)	4.04 (0.82)	4.02 (29)	<0.001	0.360	0.310	0.073

^a^ Paired *t* test.

^b^ The effect of engagement profile (average engagement vs. high engagement) on the outcome at follow-up controlling for outcome at baseline.

## 4. Discussion

We employed a person-centered approach to analyze engagement patterns within a randomized control trial of *imi,* a web-application for LGBTQ+. Based on *overall* and *content engagement* with the app, we identified two engagement profiles: “average engagement” and “high engagement.” For *overall engagement*, most participants fell into the “average engagement” profile, while only 33% were classified as “high engagement.” We also examined the distribution of four metrics (total number of sessions, pages visited, external links clicked, and time spent) within each profile and identified correlations among them, indicating similar distributions between the metrics. For *content engagement*, we also identified two profiles (“high engagement” and “average engagement”), with the number of stress-related pages viewed most clearly differentiating the two profiles. Considering that engagement with DHIs positively predicts health outcomes, further research is necessary to explore the factors that drive participants to engage more with certain content compared to others. Understanding these drivers will enable researchers to implement content in real time that allows customization for participants' needs, thereby enhancing their engagement.

We observed a strong agreement between *overall engagement* and *content engagement*. This suggests that participants who engaged with the intervention content in a general sense exhibited similar patterns of engagement with specific content pages. However, it remains significant to examine both types of engagement profiles. Our exploration of *content-specific engagement* revealed that participants in the “high engagement” profile in *content engagement* were motivated to read the stress-related page more frequently than other pages. This suggests that *overall engagement* in terms of duration, frequency, and quantity may be influenced by pages addressing minority stress. Since our study was cross-sectional, we were unable to determine whether participants experiencing higher levels of stress were more engaged with *imi* or if the stress section was simply more attractive to engage with compared to other pages. Obtaining this information will enable researchers to develop more tailored interventions that meet the specific needs of individuals and promote effective engagement strategies (4).

The findings of this study underscore the significance of engagement in achieving positive outcomes in DHIs. Our analysis revealed a noteworthy association between *overall engagement* patterns and stress appraisals. Moreover, both the “high engagement” and “average engagement” profiles exhibited improvements in stress appraisals, with the “high engagement” profile showing greater enhancements compared to the “average engagement” profile. These results align with previous studies (8, 25), highlighting the potential of increased engagement with DHIs as an effective strategy for achieving improved intervention outcomes. This emphasizes the importance of promoting greater utilization of DHI tools to maximize their potential in supporting health and well-being. Moreover, efforts to determine the optimal dosage that facilitates desired outcomes without surpassing a threshold that may contribute to participant burden or compulsive behaviors.

Furthermore, our analysis revealed that participants in the “high engagement” profile tended to use *imi* more frequently between 6am–9am and 6pm–12am compared to those in the “average engagement” profile. This observation is closely tied to non-schooling time, indicating that engagement patterns are heavily influenced by LGBTQ+ youth who can comfortably use *imi* during periods outside of school hours. This difference in the time of engagement between profiles highlights the need to adopt precision health approaches to DHIs to promote health based on a better understanding of individual circumstances. For instance, DHIs could allow users to customize their preferred usage times based on when they feel most comfortable using them, and receive reminder SMS to encourage engagement during those times. Furthermore, future qualitative research could delve into understanding the underlying factors that contribute to the higher usage of certain hours by “high engagement” users compared to “average engagement” users.

Our approach to conceptualizing engagement using alternative statistical methodologies has improved our understanding of engagement by recognizing that four domains in *paradata* metrics contribute to the level of engagement simultaneously. Traditionally, researchers have employed single *paradata* metrics independently, assuming that each metric independently influences the level of engagement, even when these metrics are highly correlated (8, 17). In the original analysis of the *imi* RCT data, individual *paradata* metrics such as session count (≥5 sessions), duration (>10 min), or page views (>10 pages) were associated with improved outcomes among participants in the *imi* intervention group (25). These findings align with our study results, which indicate that participants in the “high engagement” profile were more likely to demonstrate greater improvements in stress appraisals compared to the “average engagement” profile. While our person-centered approach adds extra nuance and context, it is worth noting that the results observed in our study were not significantly different from those in the original *imi* paper. This comparison underscores that, despite their greater comprehensiveness, person-centered approaches can still yield similar conclusions to the methodologies that examine measurements of individual metrics, thus demonstrating the robustness of the findings.

Furthermore, our study contributes to researchers' understanding of different levels of engagement thresholds. Determining the minimum engagement threshold required to induce behavioral changes has been challenging for researchers (17). However, this study defined levels of engagement using latent profile analysis, identifying a distinct “high engagement” profile characterized by participants exhibiting a specific range of *paradata* metrics within the use of *imi*. This nuanced approach to understanding engagement can provide valuable insights for designing interventions and tailoring strategies to meet engagement thresholds.

Additionally, our study highlighted two distinct conceptual *paradata* categories: *overall engagement* and *content engagement*. It is noteworthy that although there was a high level of agreement among users in terms of these two forms of engagement, they exhibited different associations with our primary outcome. The reasons behind stress appraisals being more strongly associated with *overall engagement*, as opposed to *content engagement*, could be influenced by the limited sample size. Although there was substantial agreement between *overall engagement* and *content engagement*, we need to highlight that the baseline stress appraisal scores for the high engagement profile in *content engagement* were higher than those for *overall engagement*. The relatively small sample size of only 30 participants in the high engagement profile could impact the results. Therefore, this discrepancy in baseline scores with small sample size led to non-significant results for *content engagement*. Further research with a larger sample size and incorporating additional longitudinal analyses would be beneficial for delving deeper into any discrepancies in the results. It is also possible that the total amount of the intervention consumed was a better predictor of stress appraisals than the specific type of content consumed because all sections of the guide focused on scaffolding identity affirmation and coping with minority stress, and therefore, the degree of engagement with the different sections may not have been differentially effective.

This study has several limitations. First, we were unable to examine real-time changes in participants' behaviors based on their engagement. Although *paradata* was collected continuously during the study, the measurement of behavior and outcomes was collected at only two time points, limiting our understanding of real-time behavior changes in response to engagement. Second, while we conceptually framed the *paradata* metrics based on existing literature, there is a possibility that the metrics researchers consider most important for engagement may not align with what participants themselves perceive as most significant. For example, the way users navigate modules or components within DHIs (i.e., mouse movements or engagement sequence), though not measured in the current study, may yield additional insights and could be included as additional *paradata* metrics. Further research is needed to compare qualitative and quantitative analyses of engagement in DHI in order to determine the most impactful metrics for measuring engagement. Third, the study sample size was limited, which restricted our ability to explore more detailed engagement patterns. Future studies with larger sample sizes would be valuable in helping researchers comprehend the various nuanced engagement patterns, ultimately aiding in the promotion of effective engagement strategies. Finally, our study obtained informed consent from participants for the collection of engagement data, thereby minimizing ethical concerns related to *paradata* usage. However, it is crucial to acknowledge that the utilization of *paradata* in different contexts may entail additional ethical considerations that need to be addressed (39).

Given the influence engagement has on DHI outcomes, it is crucial to explore ways of gaining a comprehensive understanding of user engagement. This requires efforts to operationalize *paradata* both conceptually and statistically, as different approaches can lead to diverse outcomes and conclusions. In our study, we employed latent profile analysis as a statistical method to classify engagement, which proves promise in addressing concerns related to multi-collinearity of *paradata* in DHI research. Furthermore, by pooling interrelated *paradata* and examining engagement across multiple domains, we were able to identify two distinct types of users based on their engagement patterns. This holistic categorization of users can be valuable for monitoring users' compliance with recommended DHI doses or creating targeted engagement campaigns aimed at transitioning users from one category to another (e.g., shifting from average engagement to high engagement). In future DHIs, it is advisable to explore user engagement using methods such as latent profile analysis that allow for the consideration of interrelated *paradata* across multiple domains. This approach, combined with different aspects of operationalization, will enable researchers to gain a deeper understanding of the complexities associated with engagement.

## Data Availability

The data analyzed in this study is subject to the following licenses/restrictions: The data gathered during this study is not publicly available and cannot be shared due to confidentiality. Further inquiries can be directed to the corresponding authors. Requests to access these datasets should be directed to SC, skchoi@nursing.upenn.edu.
